# Validation of an Artificial Intelligence-Based Ultrasound Imaging System for Quantifying Muscle Architecture Parameters of the Rectus Femoris in Disease-Related Malnutrition (DRM)

**DOI:** 10.3390/nu16121806

**Published:** 2024-06-08

**Authors:** Sergio García-Herreros, Juan Jose López Gómez, Angela Cebria, Olatz Izaola, Pablo Salvador Coloma, Sara Nozal, Jesús Cano, David Primo, Eduardo Jorge Godoy, Daniel de Luis

**Affiliations:** 1 DAWAKO Medtech S.L., Parc Cientìfic de la Universitat de Valencia, Calle del Catedratic Agustín Escardino Benlloch, 9, 46980 Paterna, Spain; sgarcia@dawako.es (S.G.-H.); acebria@dawako.es (A.C.); psalvador@dawako.es (P.S.C.); snozal@dawako.es (S.N.); jcano@dawako.es (J.C.); 2 Investigation Centre Endocrinology and Nutrition, Faculty of Medicine, University of Valladolid, 47003 Valladolid, Spain; jlopezgo@saludcastillayleon.es (J.J.L.G.); oizaolaj@saludcastillayleon.es (O.I.); dprimoma@saludcastillayleon.es (D.P.); 3 Endocrinology and Nutrition Department, Clinical Universitary Hospital of Valladolid, 47003 Valladolid, Spain

**Keywords:** artificial intelligence, disease-related malnutrition, muscle architecture parameters, reproducibility, reliability, ultrasound imaging

## Abstract

(1) Background: The aim was to validate an AI-based system compared to the classic method of reading ultrasound images of the rectus femur (RF) muscle in a real cohort of patients with disease-related malnutrition. (2) Methods: One hundred adult patients with DRM aged 18 to 85 years were enrolled. The risk of DRM was assessed by the Global Leadership Initiative on Malnutrition (GLIM). The variation, reproducibility, and reliability of measurements for the RF subcutaneous fat thickness (SFT), muscle thickness (MT), and cross-sectional area (CSA), were measured conventionally with the incorporated tools of a portable ultrasound imaging device (method A) and compared with the automated quantification of the ultrasound imaging system (method B). (3) Results: Measurements obtained using method A (i.e., conventionally) and method B (i.e., raw images analyzed by AI), showed similar values with no significant differences in absolute values and coefficients of variation, 58.39–57.68% for SFT, 30.50–28.36% for MT, and 36.50–36.91% for CSA, respectively. The Intraclass Correlation Coefficient (ICC) for reliability and consistency analysis between methods A and B showed correlations of 0.912 and 95% CI [0.872–0.940] for SFT, 0.960 and 95% CI [0.941–0.973] for MT, and 0.995 and 95% CI [0.993–0.997] for CSA; the Bland–Altman Analysis shows that the spread of points is quite uniform around the bias lines with no evidence of strong bias for any variable. (4) Conclusions: The study demonstrated the consistency and reliability of this new automatic system based on machine learning and AI for the quantification of ultrasound imaging of the muscle architecture parameters of the rectus femoris muscle compared with the conventional method of measurement.

## 1. Introduction

Disease-related malnutrition (DRM) [1] is a prevalent health issue that poses a significant challenge in our healthcare system, affecting 20% to 50% of hospitalized patients [2,3]. Its presence can lead to increased complications and mortality risk. The Effect of Early Nutritional Support on Frailty, Functional Outcomes, and Recovery of Malnourished Medical Inpatients Trial EFFORT study demonstrated that patients diagnosed with malnutrition according to Global Leadership Initiative on Malnutrition (GLIM) criteria were at higher risk for adverse clinical outcomes [4]. This condition also raises hospitalization costs [5]. Malnutrition may be linked with other conditions such as sarcopenia, characterized by muscle mass and function loss, which was traditionally associated with aging and frailty, but in 2019, the European Working Group on Sarcopenia in Older People (EWGSOP2) raised secondary sarcopenia associated with several diseases [6]. Sarcopenia might affect up to 15% of malnourished patients and 32% of cachexic older adults, increasing the risk of complications in different patient groups [7,8]. Scientific nutritional societies advise early medical nutrition treatment for at-risk medical and surgical patients to provide appropriate Medical Nutrition Therapy and prevent potential complications. In this context, measuring muscle mass is crucial for diagnosing DRM and loss of muscle mass. Muscle ultrasound, which evaluates fat-free mass and fat mass, is an emerging technique that quantifies muscle in malnutrition [8]. It has advantages over computed tomography (CT), magnetic resonance imaging (MRI), or dual photon X-ray absorptiometry (DXA) techniques due to being cheap, portable, and non-invasive [9]. Additionally, bioelectrical impedance analysis (BIA) of muscle mass may be preferable to DXA; however, specific populations require validated prediction equations [6]. Furthermore, while DXA and BIA have cut-off values for assessing muscle quantity but not quality indexes; CT and MRI can measure both quantity and quality, although clear cut-off points are still undefined [10]. Ultrasound assessment of muscle volume, area, fascicle length, and muscle pennation angle in both transverse and longitudinal positions is a valuable clinical technique [10].

However, there remains a need for the standardization of methods and measures. The SARCopenia through Ultrasound (SARCUS) Working Group proposed anatomical landmarks for ultrasonographic muscle assessment in 2018 with guidelines on patient positioning, system settings, and components to be measured [10]. Recently updated by the SARCUS group, ultrasound’s application to measuring sarcopenia includes detailed descriptions of measurement points and muscle parameters for various muscles and muscle groups [11]. Previously, we described the standardization of ultrasound measurement of rectus femoris specifically tailored for clinical practice [9]. The main problem of muscle ultrasound is the great interobserver variability that exists. Therefore, automatic analyzing systems based on AI and machine learning algorithms can help homogenize the results obtained with muscle ultrasound. In this context, the objective of this study was to validate the use of a novel software tool for medical conventional ultrasound B-mode Ultrasound Imaging System. This automatic system is a cloud-based web application software (i.e., software as a medical device) for the visualization, quantification, and analysis of medical ultrasound images with the capability to be used with any computer and compatibility with the DICOM^®^-Digital Imaging and Communications in Medicine, the international standard for medical images and related information [12]. AI is developing fast. It is right now changing our lives by improving healthcare (e.g., making diagnosis more precise, enabling better prevention of diseases). AI is a collection of technologies that combine data, algorithms, and computing power. Advances in computing and the increasing availability of data are therefore key drivers of the current upsurge of AI [13].

As ultrasound instruments have become smaller, less expensive, and easier to use, diagnostic ultrasound has become increasingly popular among a wide variety of physicians. The ultrasound imaging technique has replaced or complemented many radiographic and nuclear medicine procedures and has opened new areas of diagnostic investigation, especially in the evaluation of patients with DRM through the study of the quality and quantity of muscle. 

Considering the importance that muscle ultrasound, and especially RF, has in nutritional assessment and the possible interobserver variability in this technique, it is important to develop automatic assessment systems that allow obtaining reliable parameters from the ultrasound images captured in real-world practice. Without a doubt, these AI analysis systems are still unknown outside of research areas, however, they will be implemented in many areas of clinical image analysis. Clinical studies are scarce, so our work attempts to validate the new system in real clinical practice.

The aim was to validate an AI-based system compared to the classic method of reading ultrasound images of the rectus femur (RF) muscle in a real cohort of patients with disease-related malnutrition.

## 2. Materials and Methods

### 2.1. Subjects

A total of one hundred consecutive adult patients with disease-related malnutrition (DRM) aged 18 to 85 years were considered eligible if they had been diagnosed (with DRM) during the visit to our Nutritional Unit and provided informed consent. 

Malnutrition was assessed by the Global Leadership Initiative on Malnutrition (GLIM) criteria [14].

The constructed dataset consisted of two sets of measurements, one corresponding to the measurements realized by a conventional method for rater 1 (i.e., method A) and the other one is the set of measurements performed by PIIXMED^TM^, rater 2 (i.e., method B). The raters were kept blinded to the initial findings, (i.e., measurements for each muscle, for each MAP parameter, and the same variable for each rater).

### 2.2. Inclusion/Exclusion Criteria

Exclusion criteria included liver dysfunction (aminotransferase levels > 3 times the upper reference limit); chronic renal failure (glomerular filtration rate < 45 mL/min/1.73 m^2^); previous Intensive Care Unit (ICU) stay during the last hospital admission; cancer patients with an Eastern Cooperative Oncology Group performance status ≥ 3 points; eating disorders; any musculoskeletal disease preventing unassisted walking ability; dementia, cognitive impairment, or any neurological/psychiatric condition that may interfere with study procedures; life expectancy of less than six months; and refusal to sign the informed consent form. 

### 2.3. Ethics Committee

The study protocol received approval from the Ethics Committee for Clinical Research of the Health Council of HCUVA (protocol code PIP23341, approval date November 2023), as well as from the individual Institutional Review Boards of the participating hospitals. Written informed consent was acquired from all patients involved in the study. 

### 2.4. Screening Process

**Figure 1:**
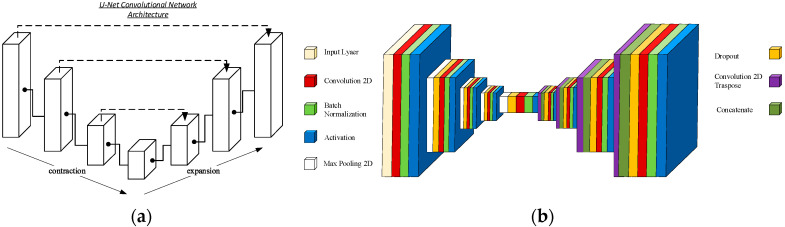
The U-net is a convolutional network architecture for fast and precise segmentation of images. Up to now, it has outperformed the prior best method (a sliding-window convolutional network). (**a**) Generic U-Net architecture; (**b**) specifically developed U-Net architecture for automatic musculoskeletal system segmentation depicting the constitutive layers in the contraction and expansion phases.

In all patients, a conventional ultrasound determination of the rectus femoris (RF) was performed by the same investigator, capturing the ultrasound images and subsequently analyzing them with the automatic system PIIXMED^TM^, (Dawako Medtech S.L., Valencia, Spain). This cloud-based web system is a convolutional neural network (CNN), with a U-net architecture, see Figure 1.

Ultrasound assessments of the unilateral (right) RF were conducted in all patients at risk of malnutrition by a skilled medical sonographer who was unaware of the clinical data and the other results of the nutritional assessment. A portable ultrasound system with a 4–10 cm linear probe (UProbe L6C Ultrasound Scanner, Guangzhou Sonostar Technologies Co., Ltd., Guangzhou, China) was utilized for anterior thigh muscle measurements while the patient lay supine with extended and relaxed knees. 

The acquisition site was located two-thirds along the length of the femur, between the anterior superior iliac spine and the upper edge of the patella. The transducer was positioned perpendicularly to minimize pressure on the muscle during measurement using excessive contact gel. 

All parameters were measured manually using the incorporated tools of the ultrasound device averaged over three consecutive measurements in the dominant leg, including the cross-sectional area (CSA) in cm^2^, the Y-axis (Transverse muscle thickness (MT)) in millimeters (mm) of the quadriceps rectus femoris muscles, and subcutaneous fat thickness (SFT) in mm. The Image JR program version 1.54 f (National Institutes of Health NIH, Bethesda, MD, USA) was used to determine echogenicity; this program is a method to treat radiological images developed by the National Health Institute (NIH). After the acquisition of the ultrasound images and the subsequent processing of these images by the PIXMED^TM^ system, the following analysis methodology was conducted.

**Figure 2:**
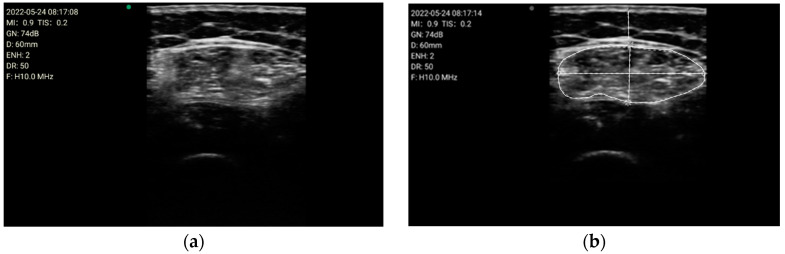
(**a**) Acquired raw ultrasound image of the unilateral (right) quadriceps rectus femoris muscle in the transverse plane measure by rater 1 (i.e., method A); (**b**) Measurement of the variables by the conventional method using the ultrasound imaging device tools, by rater 1 (i.e., method A), for the parameters of the cross-sectional area, the Y-axis, i.e., transverse muscle thickness (MT), and the subcutaneous fat thickness (SFT).

**Figure 3:**
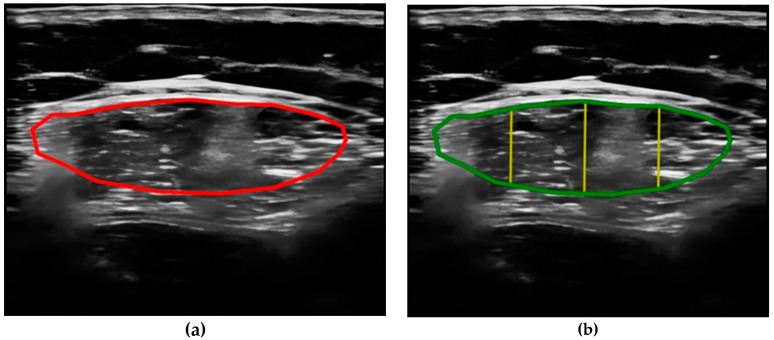
(**a**) Acquired raw ultrasound image of the unilateral (right) quadriceps rectus femoris muscle in the transverse plane obtained by rater 1, scaled and automatically segmented (red color line) by PIIXMEDTM (rater 2—method B); (**b**) PIIXMEDTM processing (i.e., rater 2—method B) of the segmented transverse ultrasound image to obtain the results of CSA (green color), and MT (three yellow lines and their mean value) parameters.

**Figure 4:**
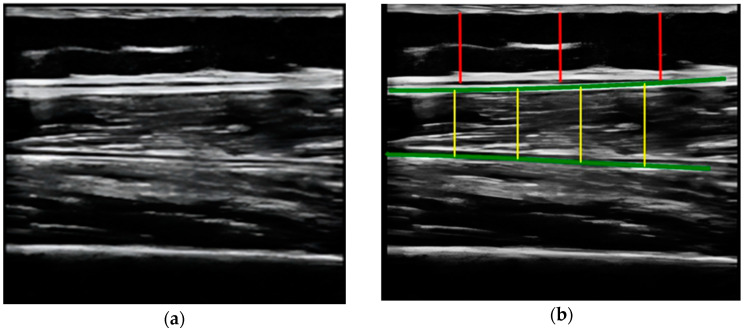
(**a**) Acquired raw ultrasound image of the unilateral (right) quadriceps rectus femoris muscle in the longitudinal plane obtained by rater 1 and scaled by PIIXMEDTM (rater 2—method B); (**b**) PIIXMEDTM processing (i.e., rater 2—method B) of the automatically segmented longitudinal ultrasound image, upper and deep aponeurosis (green color), to obtain the results of the SFT (three red lines and their mean value) parameter and the longitudinal thickness (four yellow lines and their mean value), MT, not used in this study.

1. Compare the measurements of the unilateral (right) RF of the patients performed by the expert evaluator (rater 1) using the standard tools included in the ultrasound image device (i.e., method A), see Figure 2, with those obtained by applying the PIIXMED^TM^ Ultrasound Imaging System (Dawako Medtech S.L., Valencia, Spain) (rater 2) (i.e., method B) [15,16,17,18,19] on the same acquired raw images, see Figure 3 and Figure 4.
2. Calculate and evaluate the inter-rater reliability of quantitative muscle architecture parameters (MAP) of the unilateral (right) RF measurements performed by the expert evaluator (rater 1) (i.e., method A) against the measurements using the automated PIIXMED^TM^ Ultrasound Imaging System (rater 2) (Dawako Medtech S.L., Valencia, Spain) (i.e., method B) on the same acquired raw images.

The MAP variables measured and analyzed by PIIXMED^TM^ in this study were the RF thickness and cross-sectional area in the transverse plane (MT, and CSA) and the subcutaneous fat thickness (SFT) in its longitudinal plane. 

### 2.5. Statistical Analysis

Previous statistical analysis and power and sample size determination were performed to ensure the study was adequately powered to detect meaningful effects and achieve specific statistical goals. The level of statistical power was set to 80% and a curve of sensitivity was obtained with a result of 84 subjects as the sample size. The factors for calculating the sample size were the level of 95% confidence interval, a significance level (α) of 5%, and the variability (standard deviation) of the data. 

It is important to assess the number of measurement errors by evaluating the reproducibility and reliability of measurements [20]. In the context of a study, it is important to consider other statistical measures in conjunction with assessing reproducibility (i.e., the variation in the same measurement made on a subject under changing circumstances or by different operators). 

To evaluate the magnitude of error between repeated measurements, the Coefficient of Variation (CV) was used, which is a standardized measure of the dispersion of a probability distribution [21]. The CV is a statistical measure used to assess the relative variability of a set of data points, expressed as a percentage and calculated by dividing the standard deviation by the mean and then multiplying by 100. The Coefficient of Variation is particularly useful when comparing the variability of datasets with different units or scales. It provides a standardized measure, allowing for a more meaningful comparison of the relative variability between datasets. 

After the normality of the data was assessed using Kolmogorov–Smirnov test statistics for normally distributed variables, such as MT, the correlation between method A and method B was estimated using Pearson’s (i.e., r) linear relationship. For non-normally distributed variables like SFT and CSA, the correlation between method A and method B was estimated using Spearman’s rank correlation test for association between the two variables, (i.e., ρ).

Also, Linear Regression analysis was applied to evaluate reproducibility by obtaining the percentage of the explained variation (i.e., r^2^), which represents the proportion of the variance in the dependent variable that can be explained by the independent variable in a linear model, being a measure of the goodness of fit.

Then, the Intraclass Correlation Coefficient (ICC) was used to evaluate reliability to assess the consistency or agreement under changing conditions or different raters. There are three versions of the ICC introduced in the literature depending on the experimental design and goals of the study [22,23,24]. The commonly used models of ICC are as follows: one-way random effects, two-way random effects, and two-way mixed effects. The one-way random effect was selected for the objective of this study, (i.e., assessing the reliability and consistency of measurements made by different raters or instruments on the same subjects). The classification of Intraclass Correlation Coefficient (ICC) scores varies from 0 to 1. Higher ICC values indicate greater agreement or consistency between measurements. ICC values above 0.75 are considered excellent, between 0.60 and 0.74 good, between 0.40 and 0.59 fair to moderate, and below 0.40 poor.

Together with ICC, the Bland–Altman analysis method was used [25] to assess the agreement between two measurement techniques or observers. It is a scatter plot of the differences between the two methods against their average. The analysis provides insights into the agreement, bias, and limits of agreement between the two methods. The Bland–Altman plot is widely used to visualize the difference in two continuous measurements from the same individual, graphed according to the average value of the two measures. In terms of the musculoskeletal system, this is highly valuable to assess measurements taken on the same patient by two different operators. This method can also be used for assessing two measurements made by the same operator or two measurements using different techniques or in different environments [20].

The software package used for statistical analysis and calculations was RStudio 2023.06.0 Build 421—(Posit Software, PBC formerly RStudio, PBC. The open-source data science company, 250 Northern Ave, Suite 420, Boston, MA, USA 02210 844-448-1212). RStudio is a complete, integrated software package that provides all the data manipulation, visualization, statistics, and automated reporting.

## 3. Results

### 3.1. Dataset

The database of samples in the study was made up of 100 patients (40% male and 60% female), see Table 1. All patients had nutritional DRM, with one phenotypic and one etiological criteria [15]. 

### 3.2. Summary and Descriptive Analysis

The calculation of the univariate summary statistics for all the variables in the dataset. The number of observations, mean value, standard deviation, standard error, minimum and maximum values, skewness, and kurtosis of the distributions for SFT and MT are shown in Table 2.

### 3.3. Coefficient of Variation (CV)

The CV measurements obtained using the two methods showed similar values with no significant differences in absolute values and coefficients of variation: 58.39–57.68% for SFT, 30.50–28.36% for MT, and 36.50–36.91% for CSA using method A and method B, respectively. The results are shown in Table 3.

### 3.4. Pearson and Spearman Correlation Coefficients

Table 4 shows the results of correlations between methods A and B, showing significantly higher values for very strong correlations, with 0.864 Spearman’s monotonic positive correlation and *p* (value) = 5.2 × 10^−32^ for SFT (a); 0.969 for Pearson’s linear relationship correlation and *p* (value) = 4.8 × 10^−61^ for MT (b); 0.991 Sperman’s correlation, and *p* (value) = 1.9 × 10^−86^ for CSA (c).

### 3.5. Linear Regression Analysis

**Figure 5:**
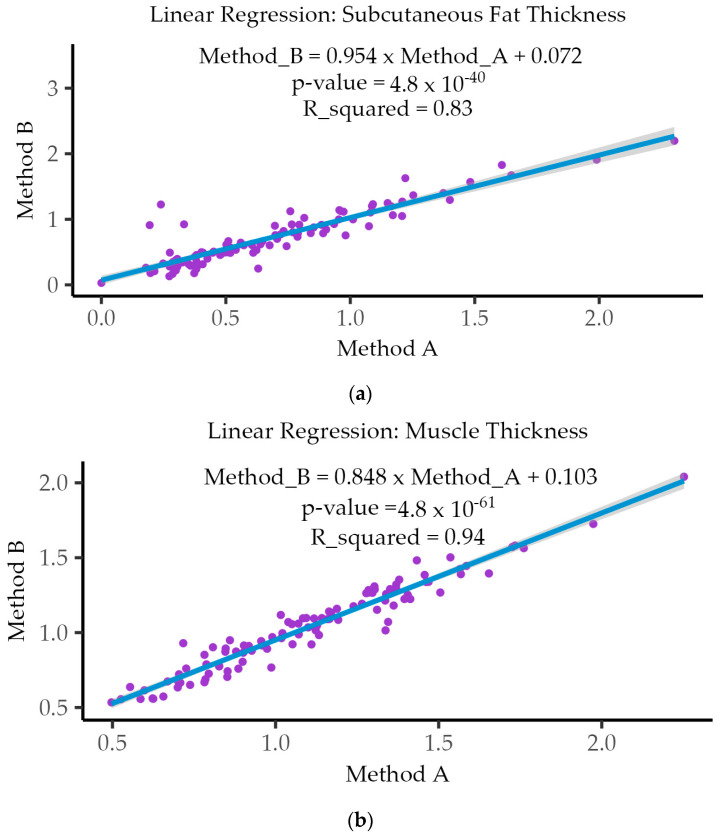

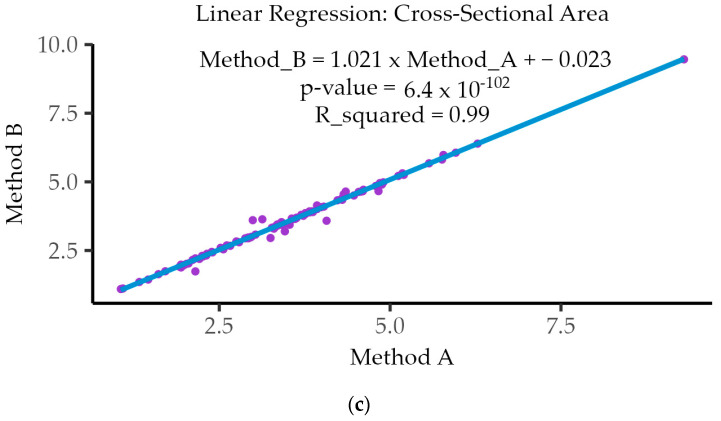
Results of the Linear Regression (r^2^) for SFT (**a**); MT (**b**); and CSA (**c**).

In the context of Linear Regression analysis, the R-squared (r^2^) value represents the proportion of the variance in the dependent variable (i.e., method B) that can be explained by the independent variable (i.e., method A) in the model. It is a measure of the goodness of fit of the regression model and describes how well one variable can be used to predict the value of the other or the strength of their relationship. Figure 5 also shows (in set) the results obtained for R-squared (r^2^) with values of 0.83 and *p* (value) = 4.8 × 10^−40^ for SFT (a); 0.94 and *p* (value) = 4.8 × 10^−61^ for MT (b); and 0.99 and *p* (value) = 6.4 × 10^−102^ for CSA (c).

### 3.6. Intraclass Correlation Coefficient (ICC)

The Intraclass Correlation Coefficient (ICC) results are detailed in Table 5. where the ICC coefficients are shown for the three variables (i.e., SFT (a), MT (b), and CSA (c)) under the three first columns of the table, with Excellent Reliability (ICC ≥ 0.9) indicating almost perfect agreement for the Single Fixed Raters and Average Fixed Raters.

### 3.7. Bland–Altman Analysis and Plots

It involves creating a scatter plot of the differences between the two methods against their average. The analysis provides insights into the agreement, bias, and limits of agreement between the two methods.

**Figure 6:**
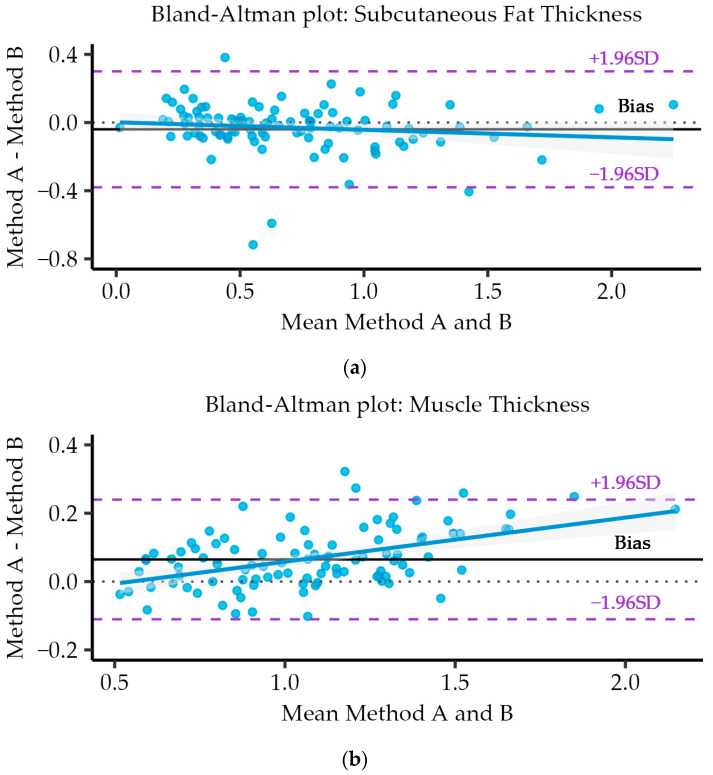

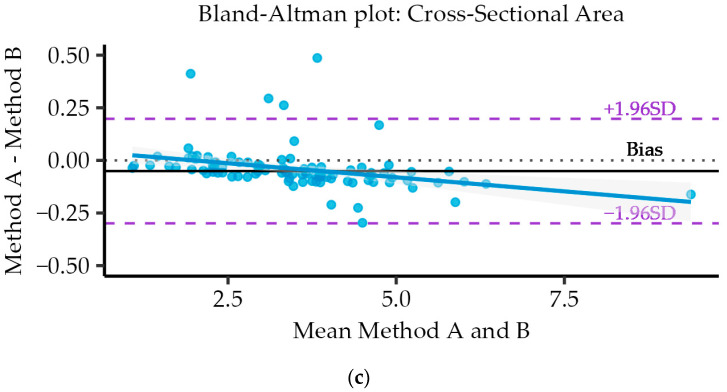
Bland–Altman analysis and plots to assess the agreement between the measurements performed by the two methods and between different measurements for each method for (**a**) subcutaneous fat thickness (SFT); (**b**) muscle thickness (MT); (**c**) cross-sectional area (CSA).

As can be seen in the plots in Figure 6, there is a consistent spread of points for the three variables (i.e., SFT (a), MT (b), and CSA (c)), with a few outliers falling outside of the LoA. These limits of agreement indicate where the true mean (and future measurements) is likely to lie. Also, the spread of points is quite uniform around the bias lines with no evidence of strong bias in any variable. In the case of the SFT (a)—bias = −0.04, and LoA = [−0.38, 0.30]; for MT (b)—bias = 0.065, and LoA = [−0.11, 0.24]; and for CSA (c)—bias = −0.051, and LoA = [−0.3, 0.20]. Table 5 describes all the quantitative results of the Bland–Altman analysis.

## 4. Discussion

Our study shows how the automatic ultrasound image analyzing system based on machine learning and AI can analyze ultrasound images of the rectus femoris (RF) with the same consistency and reliability as a trained sonographer.

There is an increasing focus in the research on assessing muscle mass using ultrasound. New studies indicate that measuring the area of the QRF muscle can be correlated with other factors such as fat-free mass, handgrip strength, and exercise capacity [26,27]. The clinical significance of ultrasound lies in its ability to assess muscle mass involvement in diagnosing malnutrition [26,28].

One of the significant challenges related to the effectiveness of ultrasound is in diagnosing malnutrition in various clinical scenarios. While specific cut-off points have not been determined yet, there are already publications attempting to identify the RF area with suitable sensitivity and specificity as a criterion for malnutrition. For instance, a multicenter study has established that a muscle area at the midpoint of the femur below 6 cm^2^ for men or 4.47 cm^2^ for females demonstrates adequate sensitivity and specificity in diagnosing malnutrition associated with PEW (protein-energy wasting) hemodialysis syndrome, a condition characterized by malnutrition, inflammation, and muscle wasting syndrome [27].

Despite the previously mentioned data, conventional analysis of ultrasound images of the muscle by an observer can have great variability and is also time-consuming during clinical consultation. If there is doubt, the arrival of automatic systems for analyzing ultrasound images could improve these limitations. 

Our automatic system based on machine learning for the visualization and automatic analysis of medical ultrasound images is a cloud-based diagnostic aid tool referred to herein as a biomarker identification system for the generation, processing, and reporting of biosignal biomarkers and quantitative ultrasound image biomarkers. The cloud-based web system is a convolutional neural network (CNN) with a U-net architecture designed for the automatic segmentation of regions of interest (ROI). The U-Net receives images as input and returns segmentation maps as output. This architecture has been developed by the department of computer science at the University of Freiburg [29]. 

The network architecture for this study was designed to work with fewer training images and produce more accurate segmentations than previous proposals. The processing algorithms are based on the open-source Python package for the extraction of features and image biomarkers from medical imaging (i.e., Radiomics) [30]. Radiomics is a rapidly developing field of research focused on the extraction of quantitative features from medical images, thus converting these digital images into minable, high-dimensional data, which offer unique biological information that can enhance our understanding of disease processes and provide clinical decision support [31,32]. 

Our automatic system supports feature extraction in 2D for conventional B-Mode ultrasound imaging and can be used to calculate single values per feature for a region of interest (i.e., segment-based). From the features identified in the images and the application of the different algorithms, diverse biomarkers are extracted and processed to analyze, among others, the anatomical measures, the mean echogenicity of the region of interest (ROI), the muscle quality based on histogram analysis of echogenicity, the texture, and other non-linear algorithms like fractality (i.e., fractal dimension). 

These biomarkers are automatically integrated into a structured report together with the results of the analysis to assist the physician in the diagnosis and assessment of a patient. Other automatic analyzing systems in ultrasound, based on machine learning and AI, are developed for pathologies such as breast cancer [33,34] or thyroid nodule characterization [35], generating in these pathologies an improvement in the speed of diagnosis and the accuracy of the prognoses compared with traditional methods. An example in nutrition is the evaluation of sarcopenia in patients with hepatocellular carcinoma [36].

To date, there is no automatic machine learning system that has evaluated muscle mass in patients with malnutrition related to disease, this being the first work to demonstrate its consistency and reliability in a pathology such as DRM with a high prevalence in our area [37]. 

Our study has some limitations. Firstly, it has only been conducted in patients with malnutrition related to disease, therefore it can only be generalized to patients with this pathology. Secondly, it was conducted in a single center, and there may be some selection bias. Thirdly, although the use of US is not a widespread technique in the determination of muscle mass in patients with DRM, clinical guidelines recommend its use [14] and a recent study has shown a good correlation with CT as a gold standard technique [38]. Fourthly, only one muscle has been evaluated, the RF. Finally, this automated method should be also replicated (and cross-validated) with another cohort and against MRI/CT [39,40] However, it also has strengths: the determination of the RF ultrasound image was perfectly standardized and only one researcher performed the ultrasounds on all patients.

## 5. Conclusions

Our study demonstrated the consistency and reliability of our new automatic system based on machine learning and AI for visualization and an automatic analysis system for the quantification of ultrasound imaging of the rectus femoris muscle compared with a conventional analysis by ultrasound in patients with disease-related malnutrition. These findings should be reproduced in future studies with a larger sample size and using other muscle groups. Without a doubt, this automated ultrasound image analysis system based on machine learning can help in the assessment of muscle mass in patients at risk of malnutrition and in patients with other entities.

## Figures and Tables

**Table 1:** Parameters of patients with DRM.

Parameters	
Age (years)	56.9 ± 16
Weight (kg)	55.6 ± 14.7
BMI (kg/m^2^)	20.9 ± 4.3
Sex (male/female)	40/60

**Table 2:** Summary and descriptive statistical analysis of the dataset.

	Subcutaneous Fat Thickness (SFT)		Muscle Thickness (MT)		Cross-Sectional Area (CSA)	
	Method A	Method B	Method A	Method B	Method A	Method B
N	100	100	100	100	100	100
Mean	0.70	0.74	1.10	1.04	3.47	3.52
SD	0.41	0.42	0.34	0.29	1.27	1.30
Min	0.00	0.03	0.50	0.53	1.06	1.10
Max	2.30	2.20	2.25	2.04	9.30	9.46
Skewness	1.21	0.93	0.54	0.42	1.00	0.97
Kurtosis	1.94	0.78	0.27	0.11	3.08	2.96
SE	0.04	0.04	0.03	0.03	0.13	0.13

**Table 3:** The table shows the CV for the distributions of method A and method B for each of the MAP variables.

Coefficient of Variation (%) Method A and Method B			
Method	Subcutaneous Fat Thickness (SFT)	Muscle Thickness (MT)	Cross-Sectional Area (CSA)
A	58.39	30.50	36.50
B	57.68	28.36	36.91

**Table 4:** Correlations between methods.

Correlation between Method A and Method B		
Variables	Correlation	*p*_value
Subcutaneous Fat Thickness (SFT)	0.864 ⁺	5.2 × 10^−32^
Muscle Thickness (MT)	0.969 *	4.82 × 10^−61^
Cross-Sectional Area (CSA)	0.991 ⁺	1.92 × 10^−86^

* Pearson’s Correlation; ⁺ Spearman’s Correlation.

**Table 5:** Complete detail of the statistical results for the ICC and Bland–Altman analysis of the MAP variables: (a) subcutaneous fat thickness (SFT); (b) muscle thickness (MT); (c) cross-sectional area (CSA).

Subcutaneous Fat Thickness (SFT)							
ICC			Bland Altman Test				
Raters	ICC Coeff.	CI 95%	Mean Diff.	SE Diff.	CI 95% Diff.	SD Diff.	Lim. 95% Agreement
Single fixed raters	0.912	[0.872, 0.940]	−0.04	0.017	[−0.07, −0.005]	0.174	[−0.38, 0.30]
Average fixed raters	0.954	[0.931, 0.969]					
(a)							
**Muscle Thickness**							
**ICC**			**Bland Altman Test**				
**Raters**	**ICC** **Coeff.**	**CI 95%**	**Mean** **Diff.**	**SE Diff.**	**CI 95%** **Diff.**	**SD** **Diff.**	**Lim. 95%** **Agreement**
Single fixed raters	0.960	[0.941, 0.973]	0.065	0.009	[0.047, 0.082]	0.089	[−0.11, 0.24]
Average fixed raters	0.980	[0.970, 0.986]					
(b)							
**Cross-Sectional Area (CSA)**							
**ICC**			**Bland Altman Test**				
**Raters**	**ICC** **Coeff.**	**CI 95%**	**Mean** **Diff.**	**SE Diff.**	**CI 95%** **Diff.**	**SD** **Diff.**	**Lim. 95%** **Agreement**
Single fixed raters	0.995	[0.993, 0.997]	−0.051	0.013	[−0.076, −0.026]	0.127	[−0.3, 0.20]
Average fixed raters	0.998	[0.996, 0.998]					
(c)							

## Data Availability

Data are unavailable due to privacy and ethical restrictions.
